# Supplementary material for: Oncilla Robot: A Versatile Open-Source Quadruped Research Robot With Compliant Pantograph Legs
Source: Front Robot AI. 2018 Jun 19;5:67. doi: 10.3389/frobt.2018.00067 (PMC7805741; doi:10.3389/frobt.2018.00067)
Supplement: Supplementary file 6 [file Data_Sheet_1.PDF]

frontiers

Supplementary Material: Oncilla robot: a versatile open-source quadruped research robot with compliant pantograph legs

Alexander Spröwitz [1,2,*], Alexandre Tuleu [1], Mostafa Ajaoolleian [1], Massimo Vespignani [1], Rico Möckel [1,3], Peter Eckert [1], Michiel D'Haene [4], Jonas Degrave [4], Arne Nordmann [5], Benjamin Schrauwen [4], Jochen Steil [5], and Auke Jan Ijspeert [1]

*Correspondence:
Heisenbergstr. 3, 70569 Stuttgart
sprowitz@is.mpg.de

ELECTRONIC HARDWARE

This lists the printed circuit boards (PCB) of Oncilla robot, their function and setup. The robot is operated through a set of modular PCB. PCB are placed on the robot's body as shown in Fig. S9. An overview of the communication and power architecture is available in Fig. 4 (main manuscript).

The main processing unit is an off-the-shelf RB-110 embedded computer running a Linux operating system. The RB-110 is based on the Vortex86DX, a 32bit x86 CPU running at 1GHz. It executes the robot's main controllers and the Central Pattern Generators. The RB-110 directly controls the position of four servo motors (S_0 in Fig.4, main manuscript). Each RC servo motor sets the adduction and abduction angle of its leg, controlled through a pulse width modulation (PWM) signal. The RB-110 board communicates with the robot's IMU (RMG 146 9-Axis, roboard) through an inter-integrated circuit bus (I2C). The RB-110 communicates with the motor driver PCB and power board through a RS-485 bus communication interface.

RS-485 communication is implemented by a custom Simple Binary Communication Protocol (SBCP) master board (Fig. 4, main manuscript). The SBCP master board features a dsPIC33FJ128 digital signal processor (DSP) from Microchip. It converts communication packages send by the RB-110 board through a point-to-point RS-232 interface to RS-485 point-to-multipoint communication, and vice versa. The SBCP master board acts as bus master for the RS-485 bus.

The Oncilla robot is equipped with a total of four motor driver PCB (Motordriver in Fig. 4, main manuscript). Each motor driver PCB is controlling two 90 W brushless motors (M_1, M_2, Fig. 4 main manuscript) of one of four robot legs. At the core of the motor driver PCB are two A3930 motor driver integrated circuits (IC) from Allegro MicroSystems, and a dsPIC33FJ128 DSP from Microchip. The DSPs are running the local PID motor position and speed control. They further allow for control of the maximum motor currents through the A3930 motor driver ICs. The DSPs directly read the relative incremental motor encoders. Each motor driver PCB communicates with three hall-effect encoders (ME_1, ME_2, ME_3 in Fig. 4 main manuscript). Those measure absolute joint angles of the corresponding legs, through a Serial Peripheral Interface (SPI) bus. Through a separate SPI bus the motor driver PCB communicate with the

corresponding force measurement PCB. Motor driver boards generate locally filtered 3.3 V, acting as power supply for the attached sensors.

Each of the four force measurement PCB of the Oncilla robot reads up to three single axis strain sensors (F_i, Fig. 4 main manuscript). Reading of strain sensors is performed with an AD7193 IC from Analog Devices featuring a differential sigma-delta analog-to-digital converter with programmable gain.

A power board converts power from a three-cell lithium polymer battery pack and provides a 24 V (maximum 25 A) supply to drive the brushless motors, a 9 V (maximum 8 A) supply for driving the servo motors, and a 6 V (maximum 3.5 A) supply for driving the logic and other electronics of the Oncilla robot.

TABLE OF VIDEOS

Table S1. Links to videos of Oncilla robot, and Oncilla robot Webots simulation.

Content	Link
Oncilla robot trots backwards and forwards	`https://youtu.be/38pX1FBRlEA`
Oncilla robot trotting up a $4°$ slope	`https://youtu.be/c7wudgzZNkc`
Oncilla robot turning on the spot, realtime	`https://youtu.be/TH8AB1mdSoY`
Oncilla robot in outdoor environment	`https://youtu.be/A20KLlwuwTg`
Webots simulation of Oncilla robot trotting forward	`https://youtu.be/0eAhhNvKjGM`

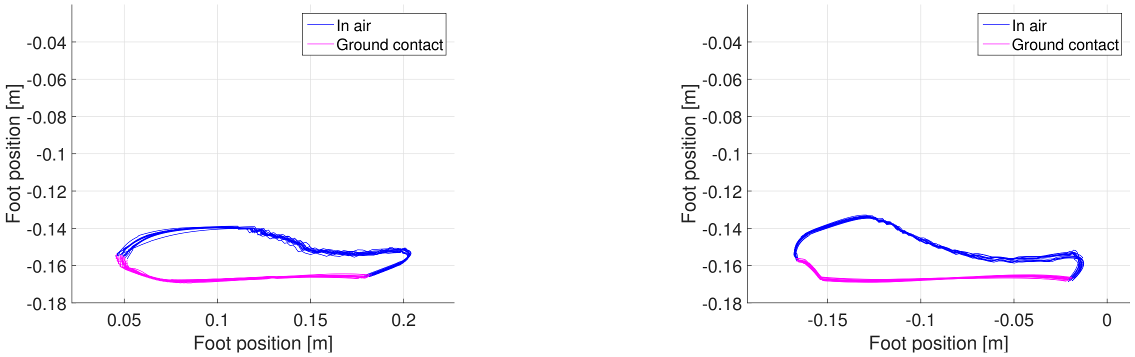

Figure S1. The recorded foot locus of Oncilla robot's Webots model, trotting at 3.5 Hz at $0.98\,\mathrm{m\,s^{-1}}$. a) the left front leg, b) the left hind leg. The commanded stride length is 0.12 m, the here observed stride length is in average 0.14 m. This is due to the foot segment, which has prolonged contact with the ground and effectively maximizes the speed of the Webots Oncilla robot model.

MOTOR OPTIMIZATION, ADDITIONAL INFORMATION

The motor and gearbox impedance matching framework by (Roos et al., 2006) was modified and applied to obtain an initial guess for the size of Oncilla robot's motors and gearboxes. The calculations were based on dynamic load estimations in the sagittal plane for the robot's leg length and leg angle motors. Adduction

and abduction (RC servo motors) load cycle scenarios were estimated based on static load scenarios.

$$P_m = P_{\text{elec}} + P_{\text{mech}} \tag{S1}$$

$$P_{\text{mech}} = (J_g + Jm)\, \ddot{\theta}_l\, \dot{\theta}_l\, n^2 + \frac{T_l}{\mu_g} \dot{\theta}_l \tag{S2}$$

$$P_{\text{elec}} = R_m\, I^2 = R_m\, \frac{T_m^2}{k_T^2} \tag{S3}$$

Motor power P_m is given in [W], the sum of electrical power losses are P_{elec}, mechanical power losses are P_{mech}. The mechanical load was defined by the externally applying torque T_l in Nm. Load angular velocity and position were given by $\dot{\theta}_l$ in $\mathrm{rad/s}$ and θ_l in rad. Inertia of moving parts of the motor were given by J_m, those of the gearbox by J_g. $\mu_g = 0.7$ is the efficiency of the gearbox. Motor winding resistance, motor coefficient, and gear ratio are given through R_m in Ohm, k_t, and n, respectively.

A second outcome from the motor optimization is a cost of transport (COT) estimation for the modeled robot (Fig. 7 main manuscript). The COT of the modeled robot (SLDM model) is compared to the real robot's COT in Fig. 7 (main manuscript). The simple model applied underestimates the real robot's COT characteristics.

NOMENCLATURE AND KINEMATIC VARIABLES OF ONCILLA ROBOT'S LEGS

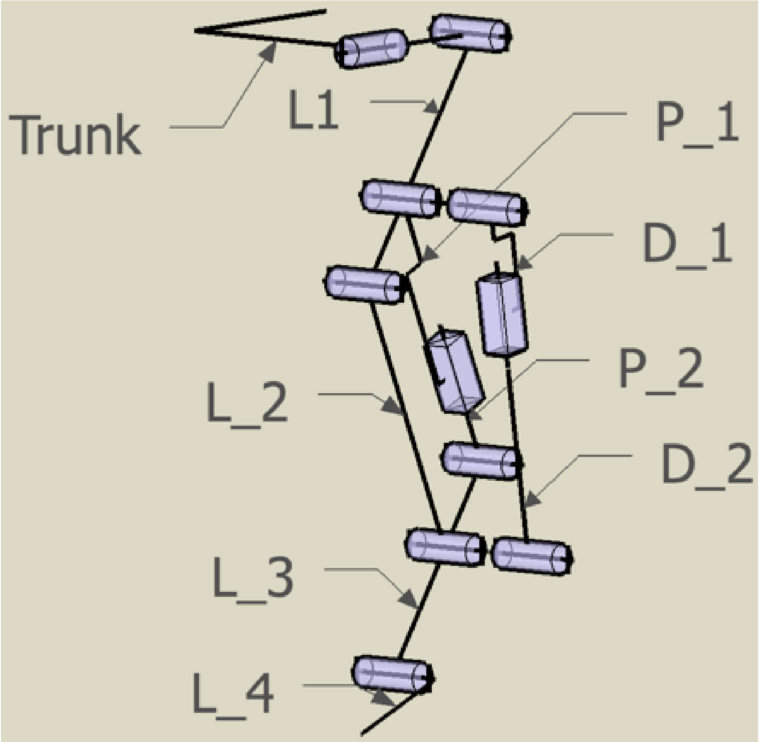

Figure S2. Oncilla robot's leg component nomenclature. Elements are numbered from proximal (close to body) to distal (towards feet). L_i are leg parts of the serial, multi-segment leg. P_i are components of the parallel strut. D_i are components of the diagonal strut. Trunk axes orientations are defined as: X forward, Y upwards, and Z sideways. The coordinate system is right hand bases.

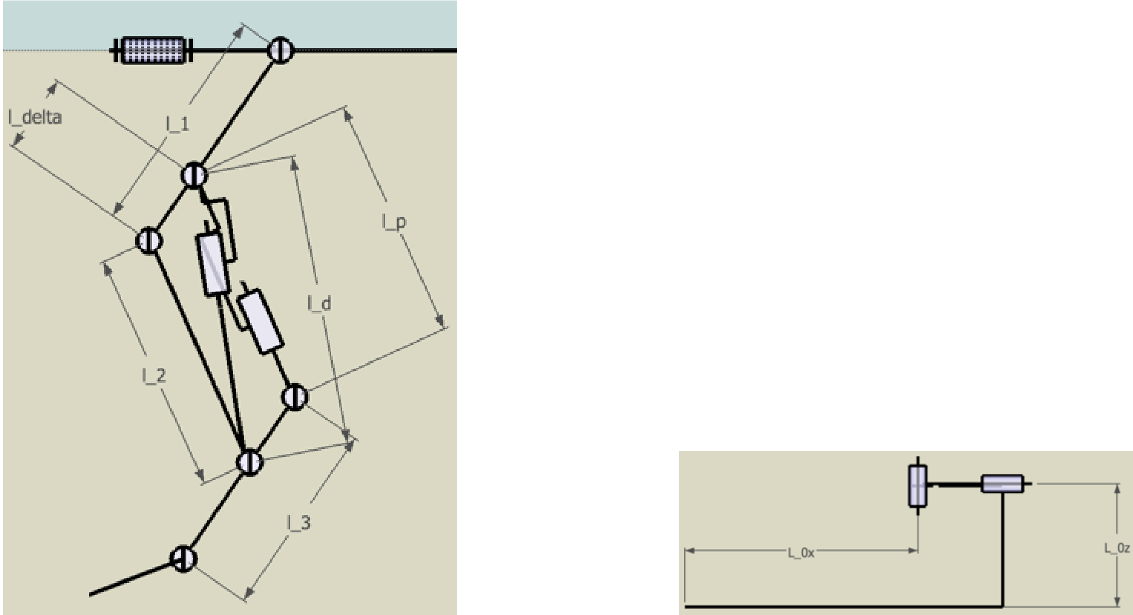

Figure S3. Leg length nomenclature. a) Side view, and b) top view.

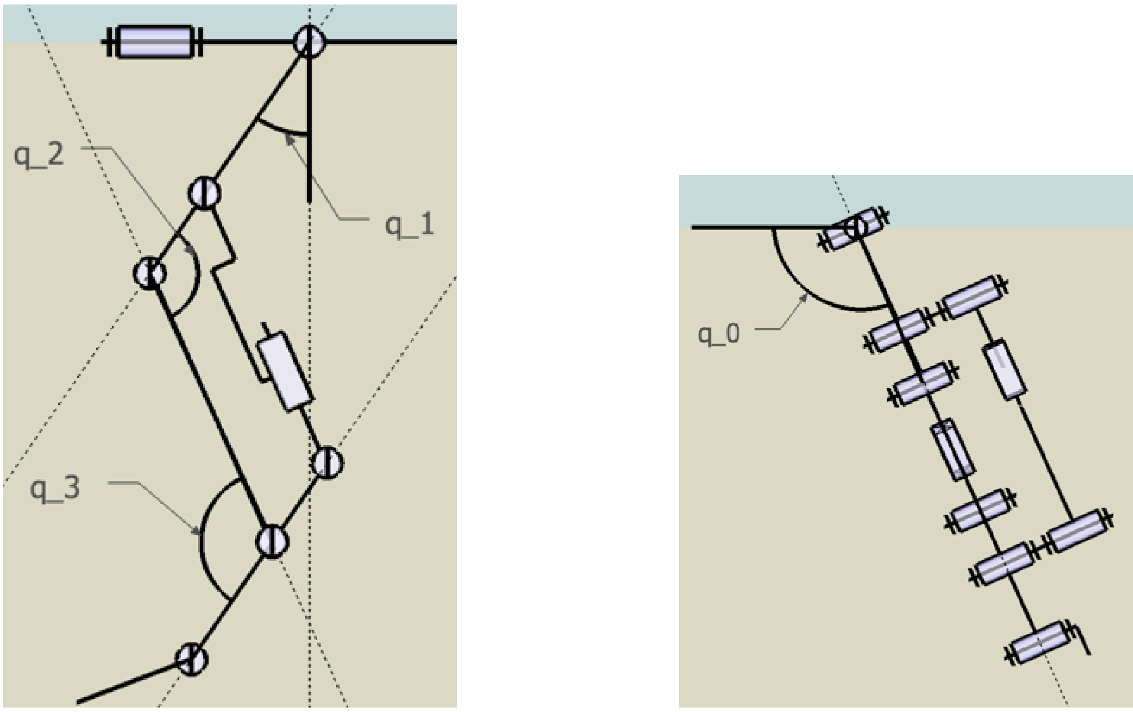

Figure S4. Definitions of leg angles in Oncilla robot. a) Side view, and b) front view.

The robot's leg elements are labeled in Fig. S2. Leg segment angles are defined in Fig. S4, and leg length definitions are provided in Fig. S3.

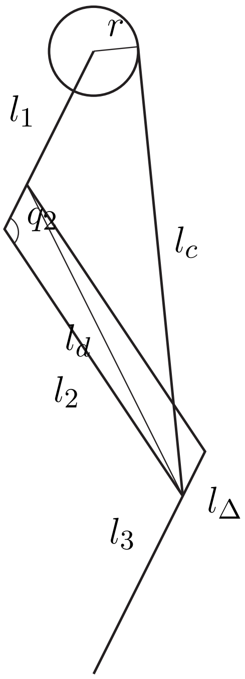

Figure S5. Nomenclature of Oncilla robot's knee mechanism.

Table S2. Kinematic variables, definitions.

q_0	Angle between trunk and L_0 segment.
q_1	Angle between L_0 and L_1, also hip angle. Motor and magnetic encoders.
q_2	Angle between L_1 and L_2, also knee angle. Magnetic encoder.
q_3	Angle between L_2 and L_3. Magnetic encoder.
q_4	Angle between L_3 and L_4, also toe angle.
$l_{0,x}$	Forward distance between the geometric center of the trunk and the q_1 axis.
$l_{0,z}$	Sideways distance between the geometric center of the trunk.
l_1	Total length of the L_1 segment, and distances between q_1 and q_2 axes.
l_2	Total length of the L_2 segment.
l_3	Total length of the L_3 segment.
l_Δ	Width pantograph: distance between q_2/q_3 and L_1-P_1/P_2-L_3 junction.
l_d	Length of the diagonal. Variable.
l_p	Length of the parallel segment. Variable.
l_c	Length of the cable, from L_0-L_1 to L_2-L_3 junctions.
r	Radius of the knee pulley.

REFERENCE POSITION AND ANGLE ORIENTATION

This paragraph describes the reference positions used both in hardware and the Webots simulation (Fig. S7). The reference position is defined by the leg length at its maximum extension, with the L_3-L_4 axis positioned vertically under the L_0-L_1 joint. Angle values and ranges are defined in Table S3.

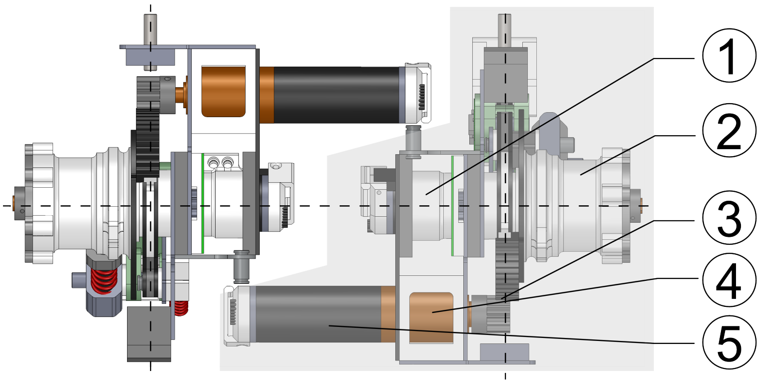

Figure S6. Top view at two front legs, CAD drawing. All other components are omitted. The right leg is indicated by an overlaid polygon. Indicator (1) is the leg length motor, covered by its ABS mount. (2) is the custom-made planetary gear of the LL motor, (3) is the custom-made spur gear pairing of the leg angle (LA) motor. (4) is the off-the-shelf gearbox (gear ratio $14:1$) of the LA motor, (5) is the LA motor. The horizontal dashed line is the hip axis, the two vertical dashed lines indicate the adduction/abduction (AA) degree of freedom.

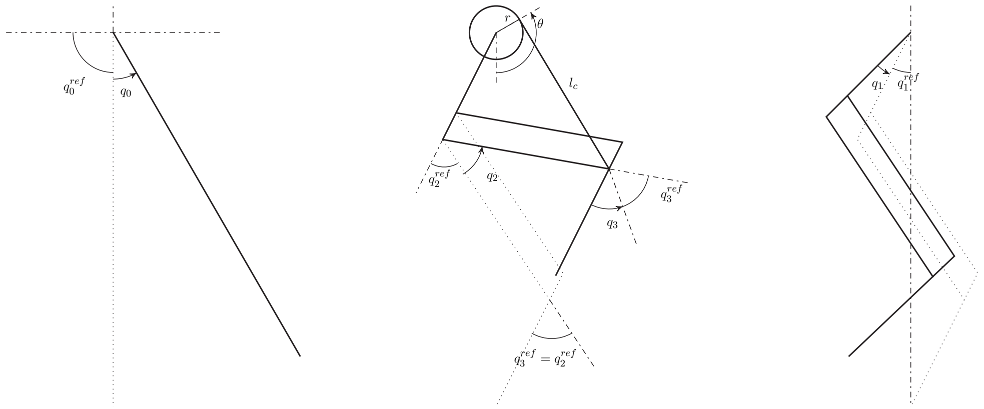

Figure S7. Robot leg reference positions. Dotted lines represent the reference leg posture. Angles with arrows are oriented in trigonometric direction. a) q_0 reference angle (front view), b) q_2 and q_3 reference angle (side view), c) q_1 reference angle (side view).

Table S3. Reference angles, and ranges.

	Fore limb					Hind limb				
	Ref	Hardware		Control		Ref	Hardware		Control	
		min	max	min	max		min	max	min	max
q_0	90°	−10°	7°	−7°	7°	90°	−10°	7°	−7°	7°
q_1	8°	−60°	65°	−50°	50°	11°	−70°	68°	−50°	50°
q_2	17°	0°	92°	0°	87°	27°	0°	91°	0°	85°
q_3	27°	0°	N.A.	N.A.	N.A.	34°	0°	N.A.	N.A.	N.A.

LEG KINEMATIC

Leg kinematic are computed based on the configuration in Fig. S7. By placing the origin at the L_0 - L_1 joint, we get:

$$x_{leg} = l_1 \sin(\bar{q}_1) + l_2 \sin(\bar{q}_1 - \bar{q}_2) + (l_3 - l_\Delta) \sin(\bar{q}_1 + q_3 - q_2) \quad \text{(S4)}$$

$$y_{leg} = -l_1 \cos(\bar{q}_1) - l_2 \cos(\bar{q}_1 - \bar{q}_2) - (l_3 - l_\Delta) \cos(\bar{q}_1 + q_3 - q_2) \quad \text{(S5)}$$

Where $\forall i, \bar{q}_i = q_i + q_i^{ref}$. Inverse kinematics of joint angles: Since q_3 is not controllable, we apply $q_2 = q_3$ for computation:

$$x_{leg}^2 + y_{leg}^2 = (l_1 + l_3 - l_\Delta)^2 + l_2^2 - 2(l_1 + l_3 - l_\Delta) l_2 \cos(\bar{q}_2) \tag{S6}$$

$$\tag{S7}$$

KNEE KINEMATICS

The goal is to find a relation between angle q_2, the knee pulley angle θ_M, and the diagonal spring length l_d. For the relation between the knee pulley angle and the cable length (under tension), and the tangent point angle θ_t the following is valid:

$$\theta_M = \frac{l_c}{r} + \theta_t \tag{S8}$$

A reference angle θ_M^{ref} was introduced, to control θ in the range of $\left[0, \theta_M^{max}\right]$. By applying the law of cosines in triangles (l_Δ, l_2, l_d) and (l_1, l_2), and Pythagoras's theorem in triangle (r, l_c), we gain the two relations :

$$l_d^2 = l_2^2 + l_\Delta^2 - 2 l_2 l_\Delta \cos(\pi - \bar{q}_2) \tag{S9}$$

$$r^2 \left(\bar{\theta}_M^2 + 1\right) = l_1^2 + l_2^2 - 2 l_1 l_2 \cos(\pi - \bar{q}_2) \tag{S10}$$

The relation between l_d and q_2 is as follows:

$$l_d = \sqrt{l_2^2 + l_\Delta^2 + 2 l_2 l_\Delta \cos(q_2 + q_2^{ref})} \tag{S11}$$

$$q_2 = \arccos\left(\frac{l_d^2 - l_2^2 - l_\Delta^2}{2 l_2 l_\Delta}\right) - q_2^{ref} \tag{S12}$$

The inverse of θ_M can be found. θ_t can be separated into two angles by the triangles (l_1, l_2) and (r, l_c):

$$\tan\left(\theta_{t,1}\right) = \frac{l_c}{r} \tag{S13}$$

$$\tan\left(\theta_{t,2}\right) = \frac{l_2 \sin(\bar{q}_2)}{l_1 + l_2 \sin(\bar{q}_2)} \tag{S14}$$

Leading to:

$$\begin{aligned} \theta_M = & -\frac{1}{r}\sqrt{l_1^2 + l_2^2 - r^2 + 2 l_1 l_2 \cos(\bar{q}_2)} \\ & + \arctan\left(\frac{1}{r}\sqrt{l_1^2 + l_2^2 - r^2 + 2 l_1 l_2 \cos(\bar{q}_2)}\right) \\ & + \arctan\left(\frac{l_2 \sin(\bar{q}_2)}{l_1 + l_2 \cos(\bar{q}_2)}\right) \end{aligned} \tag{S15}$$

INVERSE KINEMATIC

This paragraph provides the robot's inverse kinematics. Joint angle q_3 is not controllable, as it is connecting segment l_3 through a passive spring segment l_p. We simplify equations (S4) to:

$$x_{leg} = (l_1 + l_3 - l_\Delta)\sin(\bar{q}_1) + l_2 \sin(\bar{q}_1 - \bar{q}_2) \tag{S16}$$

$$-y_{leg} = (l_1 + l_3 - l_\Delta)\cos(\bar{q}_1) + l_2 \cos(\bar{q}_1 - \bar{q}_2) \tag{S17}$$

$\bar{q}_2$ is related to leg length $\sqrt{x_{leg}^2 + y_{leg}^2}$:

$$\bar{q}_2 = \arccos\left(\frac{x_{leg}^2 + y_{leg}^2 - l_2^2 - L^2}{2l_2L}\right) \tag{S18}$$

We simplify $L = l_1 + l_3 - l_\Delta$. To simplify computation of q_1, it is separated into two angles: q_1^i, the angle "induced" by q_2, and q_1^d the final angle desired for the leg ($\bar{q}_1 = q_1^i + q_1^d$). Hence:

$$q_1^d = \arctan\left(\frac{-x_{leg}}{y_{leg}}\right) \tag{S19}$$

$$q_1^i = \arctan\left(\frac{l_2\sin(\bar{q}_2)}{L + l_2\cos(\bar{q}_2)}\right) \tag{S20}$$

$$\bar{q}_1 = \arctan\left(\frac{-x_{leg}}{y_{leg}}\right) + \arctan\left(\frac{\sqrt{4l_2^2L^2 - \left(x_{leg}^2 + y_{leg}^2 - L^2 - l_2^2\right)^2}}{L^2 + x_{leg}^2 + y_{leg}^2 - l_2^2}\right) \tag{S21}$$

1 FIGURES

REFERENCES

Roos, F., Johansson, H., and Wikander, J. (2006). Optimal selection of motor and gearhead in mechatronic applications. *Mechatronics* 16, 63–72. doi:10.1016/j.mechatronics.2005.08.001

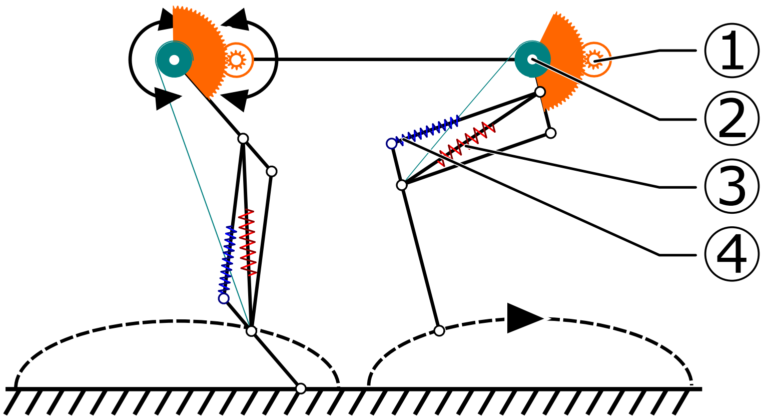

Figure S8. Schematic presentation of Oncilla robot's foot locus movement, created for the simplified-load dynamic-motor (SLDM) model scenario. The SLDM model was applied in the robot's pre-design phase, to estimate required motor and gearbox characteristics. This foot-locus profile was used to calculate leg length (LL, 2) and (LA, 1) loads, for trot gait. The diagonal, gravity compensating leg spring (red, 3) is compressed by flexing the leg through a cable mechanism. Load dependent displacement of the parallel spring (4) during stance phase was ignored in the SLDM model.

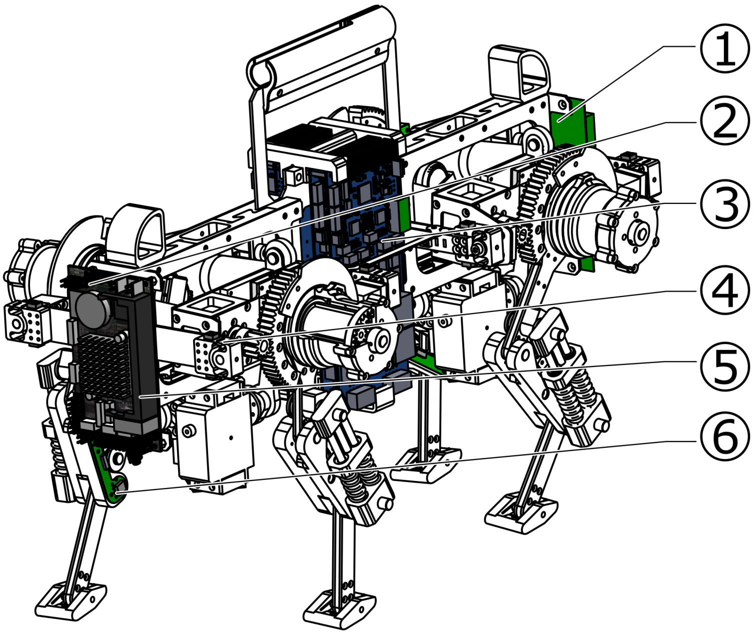

Figure S9. Indication of the placement of electronic and sensor boards on Oncilla robot. (1) The robot's power board mounted at its rear end. (2) SBCP master board, on top of the RB-110 main control board (5). (3) Motor control boards, two stacked on each side of the robot. (4) Vertical strain gage foil sensor. (6) Sensor PCB recording the absolute position of the robot's joint angles.

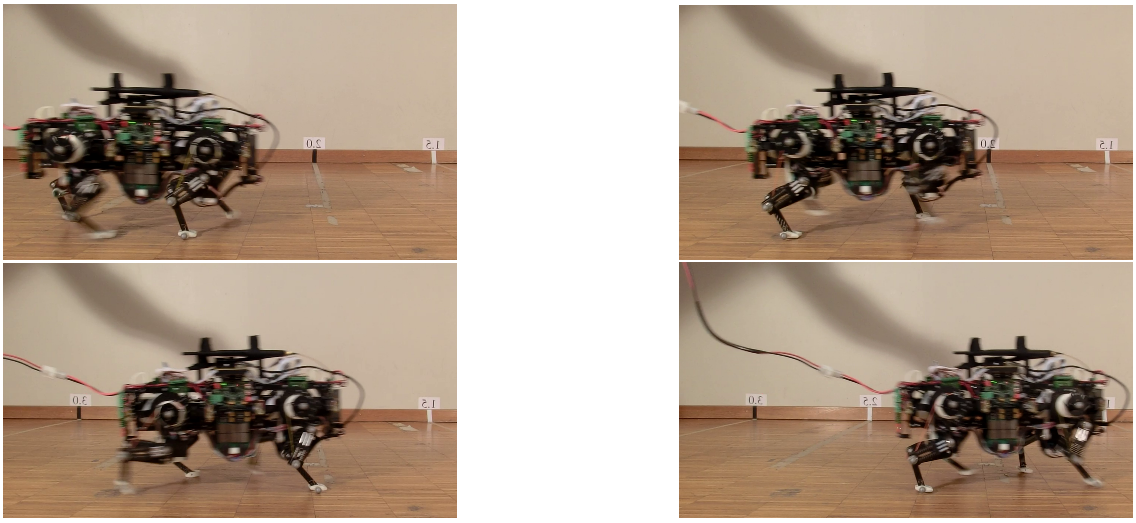

Figure S10. Hardware experiment. Snapshots of Oncilla robot during trot gait locomotion on level ground. The robot reached an average forward speed of $0.63\,\mathrm{m\,s^{-1}}$. The robot was tethered for this test, but can also locomote with on-board power. Snapshots are horizontally flipped, for reading convenience.

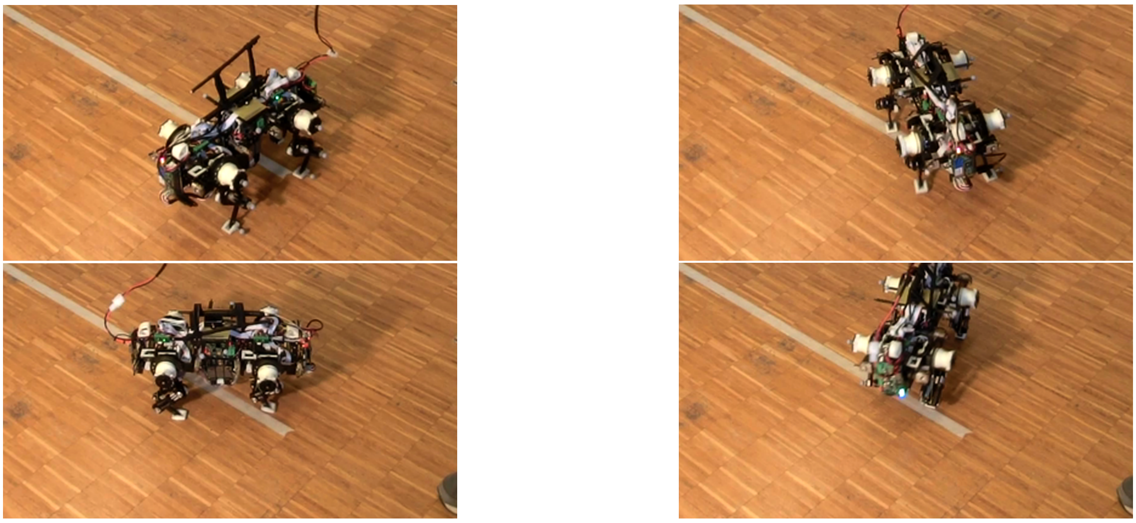

Figure S11. Hardware experiment, turning maneuver. One second is between snapshots. The robot turns $180°$, while both its sagittal motors and its abduction-adduction (AA) motors are used for turning.
